# The Impact of African Ancestry on Prostate Cancer Disparities in the Era of Precision Medicine

**DOI:** 10.3390/genes11121471

**Published:** 2020-12-08

**Authors:** Deyana D. Lewis, Cheryl D. Cropp

**Affiliations:** 1 Computational and Statistical Genomics Branch, National Human Genome Research Institute, Baltimore, MD 21224, USA; 2 Department of Pharmaceutical, Social and Administrative Sciences, Samford University McWhorter School of Pharmacy, Birmingham, AL 35229, USA; ccropp@samford.edu

**Keywords:** prostate cancer, genetics, health disparity, African ancestry, clinical trials

## Abstract

Prostate cancer disproportionately affects men of African ancestry at nearly twice the rate of men of European ancestry despite the advancement of treatment strategies and prevention. In this review, we discuss the underlying causes of these disparities including genetics, environmental/behavioral, and social determinants of health while highlighting the implications and challenges that contribute to the stark underrepresentation of men of African ancestry in clinical trials and genetic research studies. Reducing prostate cancer disparities through the development of personalized medicine approaches based on genetics will require a holistic understanding of the complex interplay of non-genetic factors that disproportionately exacerbate the observed disparity between men of African and European ancestries.

## 1. Introduction

**Figure 1:**
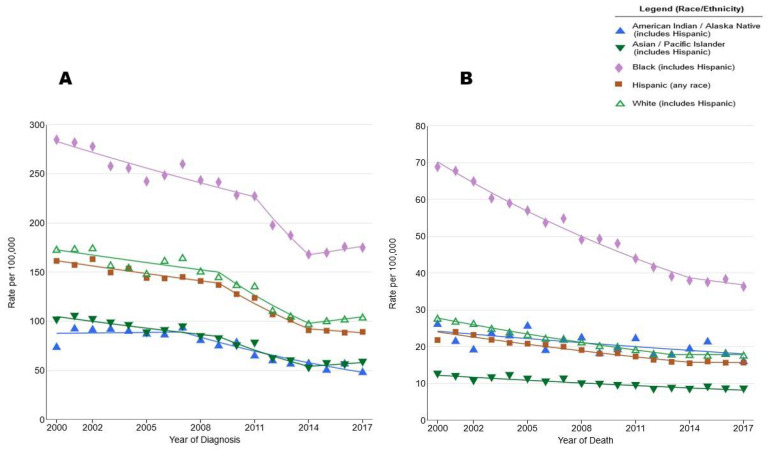
Age-adjusted prostate cancer (PCa) incidence (**A**) and mortality rates (**B**), 2000–2017. Surveillance Research Program, National Cancer Institute. SEER Incidence and U.S. Mortality Statistics (https://seer.cancer.gov/explorer/) 2020 [15].

**Figure 2:**
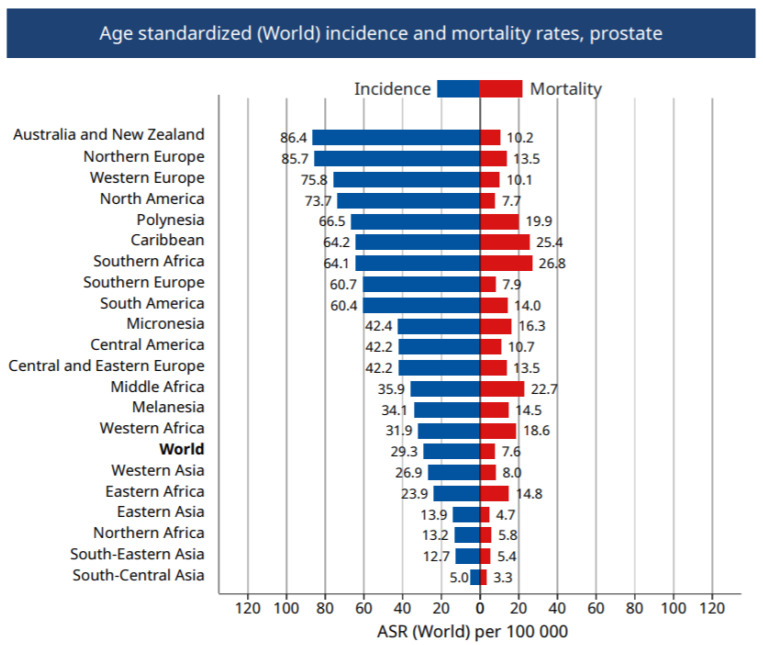
Age standardize global prostate cancer incident and mortality rate by world regions, 2018 [16].

In many developed countries, prostate cancer (PCa) is the second most frequently diagnosed cancer and is the fifth leading cause of cancer death in men worldwide. In 2018, there were an estimated 1.3 million newly diagnosed cases of PCa and 359,000 associated deaths worldwide [1]. Despite the higher burden of PCa incidence in developed countries, a disproportionate share of this burden is experienced by men of African ancestry (MAA) (Figure 1). It is well established in the literature that being of African ancestry is one of the risk factors for PCa in addition to family history and advancing age. PCa mortality rate is the highest in the world for men residing in the Caribbean and Sub-Saharan Africa (Figure 2) [2,3]. According to the International Agency for Research on Cancer (IARC), PCa is further expected to trend upward in Africa from approximately 28,000 deaths in 2010 to a little over 57,000 by 2030 [4] However, the burden of PCa incidence and mortality rates in Africa and the Caribbean could be drastically underestimated due to underdiagnoses or under treatment, instability of health management information systems, limited resources of cancer registry data, and lack of screening [5,6]. In the United States alone, PCa is the second leading cause of death among MAA, of which an estimated 29,500 cases of PCa are expected to occur among MAA in 2019, accounting for 30% of all cancers diagnosed in this ethnic group [7,8]. The average annual PCa rate for MAA between the years of 2011–2015 was 76% higher compared to non-Hispanic white men. In addition, the mortality rate among MAA men with PCa is more than two times that of their non-Hispanic white counterparts [9]. Despite advances in research, treatments, and prevention measures for PCa, MAA are still most likely to develop PCa at an early age, die from PCa, and develop more aggressive forms of the disease [9,10,11,12]. The noticeable differences in disparities among MAA worldwide are not well understood. The complex interplay of genetics, dietary, environmental, lifestyle, and socioeconomic conditions are believed to play a role in PCa disparities [13]. However, the specific causal factors remain unclear [14]. Currently, the only well-established risk factors for PCa are age, race, and family history of the disease [9]. Elucidating the causes of these disparities will be essential in the advancement of precision medicine and improving the survival outcomes for PCa in MAA.

In this review, we discuss and highlight the biological, environmental, and social risk factors in MAA in the context of the existing PCa disparity between these men and men of European ancestry (MEA). We also explore some of the implications and challenges pertaining to the lack of MAA in clinical and genomic research that could potentially play a part in exacerbating PCa disparities.

## 2. Genetic Influential Factors for Prostate Cancer Risk

### 2.1. Rare Variants of Moderate to Large Effects

Variation in genetic risk factors across ethnic groups has been studied and is increasingly recognized as one of many potential explanatory factors that may be associated with PCa disparities. Although socioeconomic conditions, lifestyle, and access to health care have been known to contribute to this disparity, after adjusting for these factors, a racial disparity in PCa for MAA remains. Therefore, a possible role for biological determinants should be explored. It has been well established that individuals with a family history of PCa experience a 2.5-fold increase in risk with a single affected first-degree relative and a five-fold increase with two or more affected first-degree relatives [17,18,19,20]. Additionally, PCa exhibits the highest reported heritability of any major cancer and the genetic contribution detected in genetic studies is approximately 58% [21]. However, the ability to identify PCa susceptibility genes has been limited.

Previous family-based linkage studies that focused on identifying loci with rare high-penetrance variants that increase PCa risk have mainly been carried out in populations of European descent. These studies yielded several genes responsible for hereditary prostate cancer such as *HOXB13* [22,23,24,25], *HPC1*(1q24-25) [26,27,28,29], *HPCX*(Xq27-28) [30], *HPC20*(20q13) [31], *PCAP*(1q42-43) [28,32,33,34], and *CAPB*(1p36) [28,33], among others [35]. Among these loci (Table 1), MAA families have shown evidence of linkage to *HPC1* [26,29,36], *PCAP*, *HPC20*, and *HPCX* [36]. Additionally, linkage signals have been identified in MAA families with a strong history of PCa for other genetic loci including 2p16, 12q24 [37], 2p21, 11q22, 17p11, and Xq21 [38], among others. However, the causal genes have not been identified for several of these linked regions. Although rare and moderate to highly penetrant risk variants other than *HOXB13* (e.g., G84E mutation) are known, most of the familial risk remains unexplained. Other studies have demonstrated predisposition genes *RNASEL*, *MSR1*, and *ELAC2* to harbor low to moderate penetrance risk alleles that have been associated with PCa and disease severity [39,40,41,42,43,44,45].

As with most cancers, diagnosis at an early age of PCa is an important indicator that suggests inherited susceptibility. For example, men with early onset PCa are most likely to harbor SNPs associated with PCa and rare mutations such as *HOXB13* G84E [23]. A recent study using data from the Surveillance, Epidemiology and End Results (SEER) program reported that 5- and 10-year relative survival rates in men with early-onset PCa were significantly worse for MAA compared to MEA (*p* < 0.0001) [46].

Studies of PCa as a multi-cancer syndrome has led to the discovery of mismatch repair genes that confer a high risk of PCa when mutated. Germline mutations in mismatch repair genes *BRCA1*, *MLH1*, *PMS2*, *MSH2*, and *MSH6* have been implicated in having an increased risk of PCa [47]. It has also been described that mutations in the *BRCA2* gene involved in hereditary breast and ovarian cancers also confers an increased risk of PCa and have potential clinical relevance regarding its association with PCa. Recent studies demonstrated that germline mutations in *BRCA1* and *BRCA2* can be essential in identifying men with higher risk of developing PCa, and are associated with a more aggressive phenotype and poorer outcome when mutated [48,49,50,51]. A few studies have indicated that mutation frequencies in *BRCA1* and *BRCA2* may differ by ethnic and racial groups [52,53,54]. Specifically, the *BRCA2* gene may be involved in early-onset PCa in MAA [53]. This may suggest that genetic testing could possibly provide significant information regarding treatment stratification. However, more studies are needed to determine if and how mutation frequencies in *BRCA1/BRCA2* vary in MAA populations.

### 2.2. Association Studies of Common Smaller Effect Variants

Some association studies in MAA have hypothesized that genetic factors involved in the androgen pathway may contribute to PCa risk among MAA [55,56,57]. For example, a polymorphism in *CYP17*, a gene involved in the androgen pathway, was associated with increased risk of PCa among MAA if they carried the A2 allele in a meta-analysis after adjusting for age, study, prostate-specific antigen (PSA) levels, and family history [55]. Other studies have supported the association between the A2 polymorphism and in PCa risk in MAA, but not in MEA [58,59,60,61]. However, one study among MEA suggested an association between the A2 allele and increased susceptibility to PCa risk in first degree relatives [62]. Although *CYP17* has shown promising results, it lacked consistent validation in case control studies across different ethnic populations [61,62,63,64,65,66]. Additionally, gene EphB2 on chromosome 1p was found to be associated with increased PCa risk in MAA with a positive family history, but this association was not found in MEA [20].

It has been demonstrated in the literature that MAA are typically diagnosed with PCa at an early age, tend to have a higher grade, and more advance stage of the disease compared to MEA [67,68,69,70]. One hypothesis that may partially explain these racial differences is that MAA generally have a shorter CAG repeat sequence on the androgen receptor (AR) gene (chromosome Xq11-12) compared to MEA [71,72,73,74]. The length of CAG repeats in MAA with PCa appears to be inversely correlated with transcriptional activity on the androgen receptor, thus yielding higher androgenic activity that may contribute to the risk of advance PCa disease [75,76]. An earlier case and control study of 1175 men discovered that men with PCa were 1.5 times more likely to have a shorter CAG repeat sequence (<19 repeats) compared with men who did not exhibit the disease (>25 repeats). This study also found that men who presented shorter CAG repeat sequences were twice as likely to have a higher grade of cancer, distant metastases, or a local spread of PCa beyond the prostate boundaries [77]. However, conclusions vary with the association between the differences in AR CAG sequence length repeats and PCa risk [78,79,80,81].

Over the last decade, technology and study designs have evolved for identifying genetic risk factors of complex disease such as PCa, which led to the discovery of approximately 170 common risk variants through genome-wide association studies (GWAS) [82]. These PCa susceptibility variants account for approximately 38% of familial risk in populations of European and Asian descent [83,84,85,86,87,88]. The common denominator of these studies is that the majority of them have been conducted in populations of European descent. As a result, progressive steps have been made in the effort to identify PCa risk variants/loci specific to African ancestral populations.

More recent studies in non-European cohorts have supported evidence that some risk variants are more common in MAA than in other ethnic/racial populations [67,89,90,91,92,93,94]. For example, the 8q24 PCa susceptibility region seems to harbor risk variants of different effect sizes that are specific to MAA including Ghanaian, South African, Afro-Caribbean and Tobago men, Ugandan, and MAA [10,92,95,96,97]. Thus far, only a few loci have been identified that exhibit genome-wide significant association in MAA. Locus 17q21: (rs7210100, odds ratio per allele = 1.51, *p* = 3.4 × 10^−13^) reached the level of genome-wide significant association in an African descent population [90] and was subsequently validated in populations of European decent [51,98,99]. More recently, a GWAS meta-analysis identified two novel genome-wide significant association signals on chromosomes 13q34 and 22q12 with the risk-associated alleles found only in MAA: 13q34, rs75823044 (OR 1.55, 95%, CI 1.37–1.76, *p* = 6.10 × 10^−12^), and rs78554043 on 22q12.1 (OR 1.62, 95% CI 1.39–1.89, *p* = 7.50 × 10^−10^) [100]. One particular GWAS that consisted of a small sample of Ghanaian men did not reach genome-wide level significance, but yielded a novel locus at 10p14 that was specific for high and low-risk PCa [101]. So far, one of these findings has been replicated. A case control study of PCa among Ugandan men resulted in nominally statistical associations and a similar effect size to those reported in the original study for 13q34 locus; rs75823044: OR = 2.02, *p* = 0.04; rs78554043: OR = 1.53, *p* = 0.44 [102].

Although the majority of PCa risk variants have been discovered in European ancestry populations, there have been many loci that harbor common risk alleles shared across most population settings [94,103,104,105]. Many susceptibility loci discovered in European and/or Asian descent populations have shown limited replication in populations of African ancestry and displayed lesser magnitude of effects or opposite directional effects by race [103,104,105]. However, Chang et al. [106] identified significant associations in MAA that were in the same direction and of similar magnitude as those reported in MEA for SNP rs10486567 at *JAZF1*, rs10993994 at *MSMB*, rs12418451 and rs7931342 at 11q13, and rs5945572 and rs5945619 at *NUDT10*/11. As of now, there have been several studies that sought to corroborate associations reported in GWAS studies of European descent populations in populations of African ancestry. One study validated PCa susceptibility variants rs7008482 (8q24; *p* = 2.45 × 10^−5^), rs6983267 (8q24; *p* = 4.48 × 10^−7^), and rs10993994 (10q11; *p* = 1.40 × 10^−3^) in South African men (331 cases and 178 controls) [10] Hooker et al. The authors in [105] validated previous GWAS SNPs on loci: (8q24; *p* = 1 × 10^−4^), 11q13.2 (*p* = 0.009), *TCF2* (17q12; *p* = 0.008), *KLK2* and *KLK*3 (19q13.33; *p* = 0.04), and *NUDT11* (Xp11.22; *p* = 0.05) in 454 cases and 301 controls of MAA. Finally, Waters et al. [104] validated *KLK2/3* (19q13.33) and *NUDT10*/11 (Xp11.22) in 860 cases and 575 controls. The loci that were validated were not consistent across studies, which could be attributed to small sample sizes in each of the studies or the possibility that PCa risk loci may differ by race, ethnicity, or geographic location. As the continuation of African ancestral specific GWAS and meta-analyses are implemented, further susceptibility variants are most likely to emerge, which may potentially explain ethnic and population differences in PCa incidence and mortality rates as well as variations of phenotypes for this disease.

### 2.3. Somatic Mutations and Tumor Biomarkers

It is important to reiterate that MAA have the highest mortality rate for PCa, however, the biology of their tumors remains understudied [107]. The overwhelming majority of tumor samples from large genomic characterization studies are from patients of European ancestry [108]. Underrepresentation of diverse racial and ethnic groups in these studies can limit the potential to detect genomic patterns and events that are enriched in these diverse groups. This can be critical when implementing patient-specific molecular targeted therapy in early stages of the disease that could improve survival for these men. Therefore, it is important to explore genetic factors that may influence tumor biology differentially across distinct ancestral backgrounds.

Recently, studies using sequence-based methods have highlighted mutational events in the biology of PCa tumors that have important biological and clinical implications. This includes the recurrent genomic rearrangements at the gene fusion product of *TMPRSS2*, an androgen-regulated transcriptional promoter, which lead to fusion transcripts and oncogenic over-expression of *ERG* (*TMPRSS2-ERG*). *TMPRSS2-ERG* fusion plays a critical role in PCa carcinogenesis, [109], occurs in over 50% of PCa patients [110,111], and has a propensity toward MEA. In contrast, MAA present PCa at more aggressive and advanced stages, however, *TMPRSS2-ERG* fusions are less frequently acquired in their tumors [112].

A growing body of evidence suggests that epigenetic changes such as DNA methylation are essential in PCa etiology [113,114,115]. One study showed statistically significant higher methylation of PCa related genes (*AR*, *RARβ2*, *SPARC TIMP3*, and *NKX2-5)* in prostate tissue samples from MAA in comparison with MEA [115]. Additional studies that highlight specific molecular aberrations in PCa of MAA include *PTEN* genomic deletions [116,117,118,119,120], differential gene expression, tumor location and recurrent deletions of *LSAMP* [116,117,119,121,122,123], *SPOP* mutations [123,124], differential expression in *SPINK1* and dysregulation of *GOLM1* loci [116,123], and loss-of function mutations *NKX3-1* [116,123,125]. Overall, the literature reveals that significant variations exist at a molecular level in tumors of MAA compared to men of European ancestry. However, the sample sizes remain small and additional larger studies are needed. In an era of PCa precision medicine, these findings have broader implications toward understanding the genomic characterization and potential discovery of novel biomarkers when MAA are included in PCa genomic studies.

## 3. Diet and Anthropometric Risk Factors for Prostate Cancer

### 3.1. Diet

While there are biological explanations for some of the differences in PCa presentation and outcomes between men of African and European ancestry, additional underlying factor such as diet can play a contributing role in exacerbating these disparities. To elucidate the contribution of environmental factors such as diet in PCa etiology, migration studies have suggested that men from areas with low PCa incidence are more inclined to acquire the incidence rate of their host country due to changes in environment [126,127]. Changes in dietary habits after migration are meaningful since the discovery that some ethnic minority populations are at a greater risk for complex diseases than the host country’s population [128,129,130,131]. Over the years, studies of PCa have examined dietary factors as an important modulator for PCa risk. Current Western dietary patterns of particularly high consumption of fat, red meat, alcohol, and dairy products may be responsible for developing a higher PCa risk. A wide range of dietary factors have been evaluated for their involvement in PCa risk, but results have remained inconclusive, [132,133]. However, epidemiological studies have shown that higher intake of processed meats, red meats, and reduction of fish may contribute to the incidence of more aggressive PCa [134,135,136,137,138,139,140], suggesting that meat intake may play an important role in PCa risk.

Very few studies have examined these associations by race. Rodriguez et al. [137] examined the association between the intake of red meat, processed meat, and poultry and its relation to the incidence of PCa among MAA and MEA in the Cancer Prevention Study II Nutrition cohort [137]. Participants consisted of 692 MAA and 64,856 MEA from the study completed a detailed questionnaire on diet, medical history, and lifestyle in 1992–1993. The follow up study in 2001 determined that meat intake was not associated with PCa risk among MEA while total red meat intake was associated with a higher PCa risk for MAA (RR, 2.0; 95% CI, 1.0–4.2, highest vs. lowest quartile). However, due to the imbalance in participant numbers within this study, it is likely that the MAA association may be false positive. One population-based case-control study showed that increased intake of foods high in animal fat was associated with PCa among MAA, but not among MEA [141]. However, a prospective cohort study did not find a strong relationship between meat intake and PCa risk in MAA than in MEA [134]. Another study evaluated the differences in dietary factors known to contribute to PCa mortality and morbidity in Nigerian men from West Africa who had migrated and were currently living in the U.S. and indigenous men living in Nigeria. This study observed that Nigerian men who had migrated and were currently living in the U.S. had a higher intake of red meat and a lower intake of fish compared to indigenous men living in Nigeria [142]. However, a higher intake of fruit and whole grain food as well as a significantly lower trans fats intake was observed in Nigerian men living in the U.S. relative to indigenous Nigerian men, thus potentially explaining a decreased PCa risk in the Nigerian men living in the U.S. cohort. Although the sample size in this study was relatively small, dietary changes of increased intake of fruits and whole grain foods and lower intake of trans fats are within the scope of guidelines recommended for lowering PCa risk by the National Cancer Institute (NCI) [143].

In addition to red and processed meats, previous studies have examined poultry intake and its relation to PCa. A prospective study of men treated with radical prostatectomy for PCa showed that very high intake of poultry was inversely associated with disease progression and very high intake of eggs was marginally associated with a higher risk of high-grade disease [144]. In a small case-control study examining PCa risk of MAA and MEA, high protein intake was either inconsistently related or unrelated to PCa risk [145]. A subsequent study failed to observe any association between poultry consumption and risk of total PCa among MAA or with total or metastatic PCa among MEA [137].

Research findings remain controversial as to whether dietary fat consumption and high diet intake of different fatty acids may independently relate to PCa risk and or progression [134,146,147,148]. A meta-analysis of 13 published case control and cohort studies revealed a statistically significant association between dietary fat and PCa [149]. However, other epidemiological studies that examined the association between the two did not support a strong association [150,151]. While the majority of cancers display increased glycolysis for the requirement of accelerated cell proliferation, PCa is characterized by low glycolysis in which its cells are dependent on the oxidation of fatty acids [152]. Certain types of fatty acids have been investigated to play a possible role in PCa development and progression [146,153,154,155]. Zhou et al. [156] examined the differences in fatty acid compositions between PCa and benign prostatic tumors among pathological conditions of the disease and between MAA and MEA. Their results revealed that MAA had higher concentrations of total fatty acids with chains of 14–18 carbons than in benign prostatic tumors compared to MEA [156]. A cohort study of 1000 men treated for PCa by radical prostatectomy observed significant differences in the fatty acid composition of periprostatic adipose tissue in African–Caribbean patients compared with MEA [157]. The conclusions of other studies remain inconsistent regarding ethnic specific associations with some fatty acids and their role in PCa [134,158].

A growing body of evidence suggests that plant-based foods and their associated nutrients demonstrate a protective association with PCa risk [159,160,161,162,163]. This led to the investigation of whether plant-based diets confer a lower risk of PCa. To gain further clarity, researchers investigated the protective association of a vegan diet with PCa risk compared with subjects subscribing to a non-vegetarian diet. In this prospective cohort study of 26,346 male participants after stratifying for race, the statistically significant association with a vegan diet remained only for the MEA (HR: 0.63; 95% CI: 0.46, 0.86). Although MAA did not show any statistical significance, there was a similarity in effect size (HR: 0.69; 95% CI: 0.41, 1.18) [164].

### 3.2. Obesity

Obesity is a well-established risk factor for a multitude of adverse health outcomes and serves as a potential risk factor that might lead to the progression of PCa [165,166,167,168]. Although African Americans generally have a higher prevalence of obesity compared to non-Hispanic whites [168], the difference in prevalence between the two racial groups of men are relatively small and have inverse relationships compared to the general obesity racial trend. From 2017–2018, the age-adjusted prevalence of obesity (body mass index (BMI) ≥ 30) for MAA was 41% compared to 45% for MEA [168]. The proposed mechanism behind its involvement is that retention of excess body fat can cause hormonal shifts in testosterone, estrogen, insulin, and insulin-like growth factor (IGF)-1, which have some degree of relation to PCa [169,170,171]. Although obesity has been linked to risk of several other cancers [172,173], its association with PCa remains unclear.

The association between obesity and PCa has been inconsistent as some studies have shown increased body mass to be associated with more aggressive tumors [174,175] while others had weak or no associations at all [174,176,177]. Previous studies investigated the racial disparities of obesity and PCa, but the results remain ambiguous. A retrospective multi-institutional analysis of the radical prostatectomy of 3162 men evaluated the relationship between obesity and PCa. This study concluded that after radical prostatectomy, obesity was found to be associated with a higher grade of PCa and higher recurrence rates. Additionally, compared to MEA, MAA had higher recurrence rates and greater body mass index (BMI) [167]. Another study discovered that obesity was inversely related to PCa among MEA and unrelated to risk among MAA [178]. On the other hand, findings from a retrospective study found that obesity is a risk factor for aggressive PCa regardless of race [179].

## 4. Social Determinants of Health

It is critical to understand that social determinants of health (also known as socioeconomic status) are the landscape of which structural inequalities produce health inequalities, which in turn, can trigger or exacerbate health disparities [180,181]. These determinants are deeply woven into the very fabric of society that can be observed by the conditions in which people live, their income status, having access to quality food, health care, housing, education, and geographic locations. These factors can indirectly influence PCa risk through biological and behavioral pathways [182,183] and the ability to receive definitive treatment [183,184,185].

Socioeconomically disadvantaged populations bear a disproportionate burden of adverse health outcomes [186] including higher incidence and mortality rates of PCa [187,188,189,190]. African Americans are affected the most by this disproportionate burden [191,192,193]. According to the 2016 Census Bureau estimate [194], African Americans make up 13% (a little over 40 million) of the U.S. population, however, 21% (nine million) fell below the poverty line, which is a yearly income of $25,465 for a family of four [195]. For every one dollar of accumulated wealth that Caucasian families have, African American families have just one cent [196]. The dimensions of these systemic inequalities are beyond the scope of this review. However, a higher income and education can affect health through a cascade effect on the ability to acquire resources such as access to better quality health care [197,198], whereas residing in a food oasis area where there are no food deserts can provide access to healthier and more nutritious food [199], thus, potentially lowering PCa risk. It has been shown that unfavorable neighborhood environments can indirectly affect PCa severity through chronic stress mechanisms. Individuals residing in disadvantaged neighborhoods experience a higher degree of emotional stress that can result in multiple negative effects on the body that may be involved in the initiation of carcinogenesis [200,201,202].

It has been hypothesized that inability to access quality health care, particularly screening for detection of the disease, can lead to a more aggressive cancer at the time of diagnosis [203,204]. This led Lynch et al. [205] to investigate whether neighborhood environment associations with advanced PCa disease differed by MAA and MEA using a novel method of neighborhood-wide association study (NWAS). This approach assessed the association between 14,663 neighborhood variables from the U.S. Census with PCa aggressiveness in MAA compared to MEA. When comparing NWAS results among MAA versus MEA, there were three variables from housing, one from education, one employment and/or one transportation variable that were found to be scientifically associated with PCa aggressiveness in MAA compared to 17 socioeconomic variables that were mainly related to poverty and/or income in MEA [205]. Discoveries such as this can be helpful in large scale gene-environment studies as well as serve as additional markers when identifying predominately African American communities/neighborhoods for PCa interventions.

MAA face several social barriers that can rather be perceived as a form of social injustice that MEA may not encounter, thus contributing to poorer outcomes and risk. For example, a study examined racial disparities in delivering definitive therapy for clinically localized PCa at the facility level between MAA and MEA. The study found significant “within” hospital quality of care variation in the rates of definitive PCa therapy between MEA and MAA, with the vast majority of facilities favoring MEA [206]. Furthermore, a subsequent study found hospitals that primarily treated minority groups were associated with lower odds of receiving definitive therapy and longer time to definitive therapy despite adjustment for race [207]. In addition, there is a growing body of research that demonstrates that in comparison to MEA, MAA are less likely to be treated for PCa with similar staging of disease [208] and experience differences in treatment starting from early prognosis to eventually terminal care [184,206,209,210,211]. For example, Wang et al. [212] reported that the treatments for PCa received between 2004 and 2011 differed by race significantly. Radical prostatectomy was performed in 39.8% of MEA relative to 27.5% of MAA. Although the patients in this study had similar clinical characteristics, the treatment plan varied in different groups. A higher percentage of MAA (37.2%) received external beam radiation therapy compared to 33.1% MEA (*p* < 0.001) Furthermore, androgen deprivation therapy was received by 9.5% MAA compared to 5.7% of MEA. In addition, a greater percentage of 12.5% MEA were in watchful waiting compared to 7.2% of MEA (*p* < 0.001) [212]

Disparities in health care insurance have been correlated with poorer access to health care, which can result in worse outcomes for racial/ethnic minority populations [213,214]. African Americans are 70 percent more likely to be uninsured than Caucasians and are more likely to avoid care because of its cost [215]. Previous studies have shown that the disparity gap diminishes in cancer mortality of MAA versus MEA patients after they become eligible for universal health care from Medicare [1,216]. Similarly, studies have demonstrated that among patients with PCa, there was no observable difference between MAA and MEA with Medicaid insurance [211,216]. One notable study showed favorable outcomes for MAA compared to MEA in an equal-access medical system despite residing in areas with lower social determinants of health [217]. A more recent study sought to measure the relative importance of race compared to health care and social factors on PCa-specific mortality by using the machine learning method and random forest regression. This study used SEER data of MAA and MEA diagnosed with PCa matched by age, disease stage, and birth year, which was stratified by age and disease stage (18 groups). Results showed that while race was somewhat of an important predictor of PCa mortality, the factors associated with racial disparities (health care and social factors) were more important in all but two of the 18 groups [218].

Reports have suggested that social determinants of health are contributing factors for PCa racial disparities, however, some results remain controversial [183,219]. Evidence of nonfinancial barriers such as poor health seeking behavior have been shown to delay diagnosis of PCa among MAA. In addition, physician bias coupled with fear of PCa diagnosis and distrust of the health care system appear to be the most evident factors [220,221].

## 5. Lack of Diversity in Clinical Trials and Genetic Studies

Over the past 15 years, the number of therapeutic clinical trials have significantly grown for men with PCa. Considering the variety of PCa treatment options that continue to develop from clinical trials, it is essential to ensure that all men receive optimal therapy. However, if a subset of this population is underrepresented or excluded, important findings that could extend their life spans and/or improve their quality of life will be lacking. Historically, African Americans, along with other minorities, have been underrepresented in clinical trials (Table 2) and genetic research, which is critical for the advancement of therapeutic and research technologies [222,223,224,225]. In an effort to address disparities in clinical trial research, the United States Congress enacted the National Institutes of Health (NIH) Revitalization Act in 1993 mandating investigators to prioritize the inclusion of women and minorities in clinical trials [226]. However, since then, few clinical and biomedical research studies have focused their recruitment efforts on the inclusion of adequate minority representation [227].

Unfortunately, there continues to be a stark underrepresentation of African Americans in clinical trials despite the striking racial/ethnic disparities in PCa incidence and mortality rates [228]. A recent study collected data on PCa clinical trials in the U.S. and found that 54.7% (23/42) had no ethnic stratification. Additionally, in all trials that provided demographic information, there were 5116 (9.8%) African Americans and 41,103 (79.4%) Caucasian participants [229]. According to a study conducted by Spratt et al. [230] from 2009 to 2015 in seven trials conducted for five new PCa therapies, only 3% of participants were MAA [230]. Additionally, a recent PCa prevention/treatment retrospective study of 17 clinical trials in six countries over the past 20 years indicated a consistent under-representation of racial minorities in clinical trials. The results from the study revealed that approximately 5% of the participants consisted of MAA [223].

Furthermore, within a 25 year span, there were less than 50% of PCa clinical trials in the U.S. that reported the participation of MAA [231]. One noteworthy study that had adequate representation of MAA enrolled (30%) is the Prostate Cancer Intervention Versus Observation Trial (PIVOT) [232]. The PIVOT study compared patients with radical prostatectomy and observation for patients with localized PCa detected in the prostate-specific antigen (PSA) screening era. Although this study provided sufficient representation of MAA, there were no differences in all-cause or PCa mortality after 12 years of follow up. Another study with ideal representation was a prospective study of the anti-hormone therapy abiraterone acetate and the steroid prednisone in 100 men (50 African Americans, 50 Caucasians) with metastatic castration-resistant PCa (CRPC) [233]. A decline in PSA was used to measure responses to the therapy. PSA levels decreased at higher rates for MAA participants and stabilized for a median of 16.6 months compared to 11.5 months for MEA participants. These findings highlight the importance of adequate inclusion of MAA participants and suggests that racial determinants may play a role in the degree of response to some treatments in patients.

Proper representation of minority populations in clinical trials and genetic studies is critical in the advancement of medicine and research. Without these adequate inclusions, scientists and pharmaceutical companies are left to speculate how new therapies that were developed based on homogenous populations can improve the standards of treatment across populations from different ethnic backgrounds. The reasoning behind the lack of representation entails dissecting apart a complexity of multi-layered factors that contribute to challenges in the recruitment of African Americans in government initiatives. It is well documented in the literature that historic unethical mistreatment of African American has led to higher levels of distrust within the African American community, therefore deterring participation in research [234,235,236,237,238].

One major historical event that has led to this inherent lack of distrust stems from the Tuskegee Syphilis Study. In this 40-year study, researchers deliberately withheld treatment from MAA with syphilis in the interest of studying disease progression [238,239,240]. The suggested attitudes of distrust that stem from this study could potentially increase PCa outcome disparities by causing reluctance to enroll in trials of novel testing, treatments, and targeted therapies that can potentially reduce PCa risk and/or improve survival among these men [241,242]. The Tuskegee Syphilis Study is unlikely the primary reasoning for the lack of participation in clinical trial studies as well as the widespread mistrust of clinical research and health care systems. Rather, this reasoning stems from broader historical and personal experiences.

Another major historical event that has resulted in generations of African Americans into not trusting medical institutions was the Henrietta Lacks story. In the 1950s, physicians at Johns Hopkins Hospital used HeLa cells derived from the cervical cancer cells of Henrietta Lacks, a 31-year-old African American mother of five, without her consent, which led to important medical advances and domestic and global scientific discoveries that continue today [243]. Numerous laboratories and companies have gained financially from the use of HeLa cells without compensation to Mrs. Lacks’ family [243]. Although the Lacks family has not received profits gained from the research involving her cells, the NIH has formally apologized for this mishap in medical treatment and has put forth moral and ethical efforts to somewhat rectify what happened to Mrs. Henrietta Lacks [244].

Previously published reasons for the lack of participation due to mistrust in the medical community include experiences with racism/discrimination and devaluation, differential treatment within the health care system and previous negative interactions and abuse from research institutions [235,238,243,245,246]. To explore MAA’s attitudes toward PCa research and genomic testing, Rodgers and colleagues conducted a qualitative study that examined these topics in a geographically diverse sample of MAA and community stakeholders [245]. The results from this study regarding barriers to participate in PCa research included a lack of PCa knowledge, confusing PSA testing, health care system distrust, and misuse of personal health information. As for genomic testing barriers, research has identified a lack of terminology understanding, reluctance about receiving medical care, unfavorable attitudes toward research, and mistrust in the health care system. Facilitators of genomic testing included the value of prevention, family history, and the desire for health education [245].

There is very little literature on recruitment strategies for African Americans in research and clinical trials, particularly among MAA. However, one common thread that remained consistent throughout the literature for improving participation is building trust between the community and researchers as well as identifying gatekeepers of the community as advocates [247,248,249,250,251,252]. Without the essential element of trust from the community, it will be difficult to gain access and engage with potential participants. Other strategies include, but are not limited to, including minority investigators and staff on the research teams who identify with the population being served [253,254], acknowledgement of participant’s time and effort by offering monetary incentives [249,253,255], and employing unconventional recruiting methods such as word-of-mouth in local places (barbershops, faith-based organizations, and community health centers) [249,254,256]. One noteworthy recommendation from Oren et al. is to have journals factor in population representation aspects when assessing a study’s merit in addition to the overall results [252].

Recent efforts have been made by research groups to successfully recruit and retain large numbers of MAA in PCa studies. In 2018, joint efforts were made by the National Institutes of Health (National Cancer Institute and National Institute on Minority Health and Health Disparities) along with the Prostate Cancer Foundation to launch a $26.5 million study to investigate environmental and genetic factors associated with aggressive PCa in MAA [257]. This study is called the “Research on Prostate Cancer in Men of African Ancestry: Defining the Roles of Genetics, Tumor Markers, and Social Stress,” or RESPOND. The RESPOND study aims to enroll 10,000 MAA with PCa. This large-scale study has the potential to unravel the complex interactions of biological and non-biological factors that contribute to PCa outcome disparities as well as producing more effective interventions and development of novel treatment strategies for MAA. Given that African Americans are considerably under-represented in genetic studies [258,259], having a substantial number of African ancestral populations participate in the RESPOND study will also give the opportunity to uncover rare genetic variants associated with PCa. Another initiative that aims to address the high burden of PCa among African ancestral populations is the Men of African Descent and Carcinoma of the Prostate (MADCaP) Consortium [260]. MADCaP is a large multicenter consortium of investigators from Africa, the Caribbean, the United Kingdom, and United States who are combining their case-control studies of PCa genetic epidemiology data in the effort to better understand the African genome and its role in establishing PCa risk in African men.

**Figure 3:**
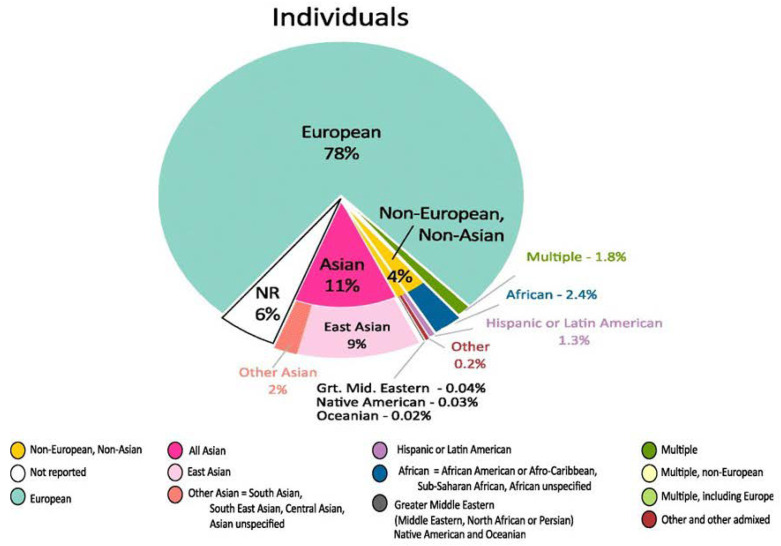
Contributions of individuals of different ancestral populations to newly discovered genome-wide association studies [259].

The lack of diversity that exists in genetic studies is not a surprising revelation (Figure 3), but it can potentially deny researchers more opportunities to discover disease-causing variants. Although inclusion efforts are improving over time, there is still a great need for genetic studies in more ancestrally diverse populations [258,259]. To ensure inclusions of diverse populations in clinical trials and genetic studies, self-reported race from subjects are frequently reported and collected, often serving as a reasonable proxy for genetic ancestry. Self-reported race errors may occur from this approach due to race often being described as a social construct and like race, how one perceives their racial identity can be fluid [261,262]. Therefore, many studies seek to focus on identifying subjects according to their geography from ancestry informative markers and appropriate statistical methods [263,264,265,266,267,268]. However, there has been controversy as to whether and how to use race and geographic ancestry in genetic and biomedical research [269,270,271,272]. Previous studies have examined the relationship between measures of self-identified race and geographic ancestry, which yielded variations of correspondence between the two [273,274,275,276,277]. Despite scientific and cultural disputes regarding race and geographic ancestry, data collection of diverse populations are warranted to better understand how measures of self-identification interact with social factors, genetic classifications, and health outcomes for all individuals regardless of how one self-identifies.

## 6. Perspectives and Conclusions

MAA have long suffered disproportionately from higher PCa incidence and mortality rates compared to other ethnic/racial groups despite advances in medicine and preventive measures. We examined the underlying factors that contribute to the racial disparity of PCa among MAA while highlighting complex multi-layered factors such as the lack of MAA in clinical trials and genomic research, and social determinants of health that can potentially play a part in exacerbating PCa disparities. The lingering effects from historic structural inequalities and events have severely affected African Americans so that an inherent wariness exists. This manifestation of distrust can in part affect participation in clinical trials and genomic studies, which in turn, can inhibit the discovery of disease-causing variants that can lead to novel PCa therapies and treatments, thus potentially extending or improving the quality of life for MAA. Although fear of exploitation based on past unethical practices serves as a partial component to the reluctance of participation in genetic/biomedical research including PCa-centered studies, research has shown that African Americans are willing to participate given the opportunity when certain criteria are met including but not limited to transparency in the agenda [245] and when objectives are translated into a culturally relevant context [278].

Perhaps the myopic viewpoints of minority/ethnic groups, specifically that of African Americans, are too difficult to reach in terms of recruitment should be reevaluated. Instead, the fact that they are hardly being reached should be considered. The understanding that the 1993 NIH Revitalization Act mandated minority inclusion, but very few mechanisms exist to enforce inclusion policies is perplexing. While scientists are often viewed as objective individuals, we are still susceptible to unrecognized implicit bias that can permeate decision-making in peer review processes [279] as well as decisions that govern funding for health disparities research [280]. Furthermore, there is an urgent need for the scientific community to reexamine its values and priorities before making decisions. Research has shown that health disparity research is less likely to be funded and can be judged as less significant and innovative compared to basic science research because of implicit bias [280,281,282]. While there is no quick solution to fix the PCa disparity that exists between MAA and MEA, the largest impact could be made by addressing the systemic structures that produce inequalities in opportunities to alleviate the disparity and achieve precision medicine equity.

## Figures and Tables

**Table 1:** Represented loci associated with prostate cancer risk.

Loci	Method	Population(s)	Gene/Markers	References ^(population)^
1p36	Linkage	European	*CAPB*	[33]
1q24-25	Linkage/GWAS	European African American	*HPC1*	[26] ^(Afr Amer)^ [28] ^(Eur)^ [29] ^(Eur)^
1q42-43	Linkage	French and German European African American	*PCAP*	[34] ^(Fra & Germ)^ [28] ^(Eur)^ [36] ^(Afr Amer)^
2p16	Linkage	African American	rs980481 and rs71527	[37]
2p21	Linkage	African American	D2S2259	[38]
11q22	Linkage	African American	D11S908	[38]
12q24	Linkage	African American	rs11067228	[37]
17p11	Linkage	African American	D17S1852	[38]
17q21	Linkage/GWAS	European	*HOXB13 (G84E)* * rs138213197*	[23]
20q13	Linkage	European African American	*HPC20*	[28] ^(Eur)^ [36] ^(Afr Amer)^
Xq21	Linkage	African American	DXS986	[38]
Xq27-28	Linkage	Finnish African American	*HPCX*	[30] ^(Fin)^ [36] ^(Afr Amer)^

Legend: Previously discovered loci of rare high penetrance loci/variants by linkage analysis or genome-wide association studies associated with PCa risk. The column headers represent information for methods, population(s), gene or markers, and references. Populations for each study are given in superscript if more than one study is presented. Afr Amer: African American; Eur: European; Fin: Finland; Fra: France; Ger: Germany.

**Table 2:** Summary of enrollment of black and white men in representative clinical trials for three Food and Drug Administration (FDA)-approved PCa drugs.

	ERLEADA^®^ (Apalutamide)	NUBEQA^®^ (Darolutamide)	AXUMIN^®^ (Fluciclovine)
FDA approval date	17 September 2019	30 July 2019	27 May 2016
Purpose of drug	Treatment of prostate cancer that has not spread to other parts of the body (non-metastatic) and no longer responds to a medical or surgical treatment that lowers testosterone (castration-resistant)		Drug for detection of prostate cancer recurrence in men who have been treated for prostate cancer but have persistently high prostate specific antigen (PSA) in their blood.
Total participants	1207	1509	596
Total black participants	68	52	26
Total black participants, %	6%	3%	4%
Total white participants	800	1194	186
Total white participants, %	66%	79%	31%
Percentage not reported	16%	4%	64%
